# Carbon isotope composition, water use efficiency, and drought sensitivity are controlled by a common genomic segment in maize

**DOI:** 10.1007/s00122-018-3193-4

**Published:** 2018-09-22

**Authors:** Viktoriya Avramova, Adel Meziane, Eva Bauer, Sonja Blankenagel, Stella Eggels, Sebastian Gresset, Erwin Grill, Claudiu Niculaes, Milena Ouzunova, Brigitte Poppenberger, Thomas Presterl, Wilfried Rozhon, Claude Welcker, Zhenyu Yang, François Tardieu, Chris-Carolin Schön

**Affiliations:** 1 Plant Breeding, TUM School of Life Sciences Weihenstephan, Technical University of Munich, Liesel-Beckmann-Straße 2, 85354 Freising, Germany; 2 INRA, UMR759 Laboratoire d’Ecophysiologie des Plantes sous Stress Environnementaux, Place Viala, 34060 Montpellier, France; 3 Botany, TUM School of Life Sciences Weihenstephan, Technical University of Munich, Emil-Ramann-Straße 4, 85354 Freising, Germany; 4 KWS SAAT SE, Grimsehlstraße 31, 37555 Einbeck, Germany; 5 Biotechnology of Horticultural Crops, TUM School of Life Sciences Weihenstephan, Technical University of Munich, Liesel-Beckmann-Straße 1, 85354 Freising, Germany

## Abstract

**Key message:**

A genomic segment on maize chromosome 7 influences carbon isotope composition, water use efficiency, and leaf growth sensitivity to drought, possibly by affecting stomatal properties.

**Abstract:**

Climate change is expected to decrease water availability in many agricultural production areas around the globe. Therefore, plants with improved ability to grow under water deficit are urgently needed. We combined genetic, phenomic, and physiological approaches to understand the relationship between growth, stomatal conductance, water use efficiency, and carbon isotope composition in maize (*Zea mays L.*). Using near-isogenic lines derived from a maize introgression library, we analysed the effects of a genomic region previously identified as affecting carbon isotope composition. We show stability of trait expression over several years of field trials and demonstrate in the phenotyping platform *Phenodyn* that the same genomic region also influences the sensitivity of leaf growth to evaporative demand and soil water potential. Our results suggest that the studied genomic region affecting carbon isotope discrimination also harbours quantitative trait loci playing a role in maize drought sensitivity possibly via stomatal behaviour and development. We propose that the observed phenotypes collectively originate from altered stomatal conductance, presumably via abscisic acid.

**Electronic supplementary material:**

The online version of this article (10.1007/s00122-018-3193-4) contains supplementary material, which is available to authorized users.

## Introduction

Predicted climate change towards higher temperatures and varying drought scenarios requires breeding adapted cultivars in order to maintain sustainable agricultural production (Challinor et al. 2014). Drought results from an imbalance between evaporative demand, originating from the atmospheric vapour pressure, and soil water availability. This generates a hydraulic signal within the plant, followed by hormonal and metabolic processes (Christmann et al. 2007; Tardieu et al. 2018). The hydraulic signal spreads rapidly from roots to leaves and vice versa (Christmann and Grill 2013; Vandeleur et al. 2014). Water uptake is controlled by the water conductance of the root system and the prevailing water potential gradient between soil and root, while water loss of leaves is primarily affected by stomatal conductance, the water potential gradient between plant and atmosphere, and the total leaf area. Water conductance in roots and leaves is regulated by the flux itself (Vandeleur et al. 2014) and the phytohormone abscisic acid (ABA; Kim et al. 2010; Caldeira et al. 2014).

By controlling transpiration rate, stomatal conductance (*g*_*s*_) defines to a large extent water use efficiency (WUE). WUE expresses plant or crop productivity per unit of water used (Rizza et al. 2012) and has been identified as one of the major components of yield under water-limited conditions (Passioura 1977). In C_3_ species, a well-established indirect trait for selection of genotypes with improved WUE and drought resistance is carbon isotope composition (δ^13^C; Farquhar and Richards 1984; Saranga et al. 1998). During photosynthesis, plants discriminate against the naturally present stable isotope ^13^C, which results in different δ^13^C in the dry matter than in ambient air (von Caemmerer et al. 2014). In C_3_ species, the discrimination mostly occurs during CO_2_ diffusion from the atmosphere to the chloroplast and during carbon fixation by the enzyme Rubisco (ribulose-1,5-bisphosphate carboxylase/oxygenase; Farquhar et al. 1982a). Here, δ^13^C has an inverse linear relationship with the ratio of intercellular to atmospheric partial pressure of CO_2_ (p_i_ p_a_^−1^) that is directly related to *g*_*s*_ (Farquhar et al. 1982b; Brugnoli et al. 1988). When photosynthetic rate is constant, reduced stomatal conductance leads to less discrimination (Ehleringer 1990) and increased δ^13^C.

In C_4_ plants, such as maize, knowledge about the association of δ^13^C and WUE is limited. The relationship of the two traits is more complicated compared to C_3_ species as C_4_ photosynthesis includes the synchronized operation of two metabolic cycles across two photosynthetic cell types (mesophyll and bundle-sheath cells) with two carbon-fixating enzymes, phosphoenol pyruvate carboxylase (PEPC) and Rubisco, playing pivotal roles. Moreover, CO_2_ leaking from the bundle sheath back to the mesophyll (bundle-sheath leakiness, *φ*) also affects δ^13^C in C_4_ species. Thus, in C_4_ plants, the correlation between δ^13^C and p_i_ p_a_^−1^ and hence *g*_*s*_ can theoretically be positive, zero, or negative, depending on the extent of leakiness (Hubick et al. 1990; Sandquist and Ehleringer 1995). Therefore, studying the genetic association of the traits δ^13^C, g_s_, and WUE and its possible effects on drought sensitivity is required for the genetic improvement of C_4_ crop species. In particular, it needs to be investigated whether the genetic control of δ^13^C is common with those of g_s_, WUE and growth.

Using an introgression library (IL), we have shown that grain δ^13^C is under genetic control in maize (Gresset et al. 2014), with several genomic segments influencing this trait. Here, we focus on a genomic segment on chromosome 7 (chr 7), where the largest effect on δ^13^C was found. The objectives of our study were to investigate to what extent variation in δ^13^C is related to relevant traits involved in drought tolerance, in particular plant growth in well-watered and water-limited conditions, plant sensitivity to evaporative demand and soil water deficit, WUE, and stomatal conductance. The high-throughput phenotyping platform *Phenodyn* (Sadok et al. 2007) allows measuring changes in leaf elongation rate (LER), the first and most sensitive trait affected by drought (Ben Haj Salah and Tardieu 1997), with high temporal resolution. In this study, we combine multiple experiments under field and controlled conditions for assessing the relationship between LER, δ^13^C, WUE, and stomatal conductance in maize.

## Materials and methods

### Plant material

Three maize lines were analysed in this study. A European elite dent line, originating from Southeastern Europe and known to perform well under water-limited conditions, served as the recurrent parent of an introgression library described previously (Gresset et al. 2014) and was used as a reference in all experiments. Two near-isogenic lines NIL A and NIL B were obtained by crossing three introgression lines described in Gresset et al. (2014; pedigree information available in Table S1, Online Resource 1). Both NILs had overlapping segments from the donor parent (a more drought sensitive flint line) in a specific region of chr 7, no overlapping segments in other parts of the genome, and maximum recurrent parent background (96.45% for NIL A and 97.25% for NIL B; Table S1, Online Resource 1). NILs A and B were developed to investigate the genetic effect of the donor segment on chr 7 on several drought-related traits in a drought tolerant genetic background.

Genotypic analysis of the NILs was performed with the Axiom™ Maize Genotyping Array (Affymetrix, Santa Clara, CA, USA). The length of introgression segments in the genomes of NIL A and NIL B was identified using Flapjack (version 1.16.10.31; Milne et al. 2010) with B73 v4 coordinates (www.maizegdb.org; Andorf et al. 2016) used as a reference.

### Field experiments

Field experiments were conducted under rain-fed conditions in the year 2014 in Roggenstein, Germany (48°10′47.7″N, 11°19′16.2″E) and in 2015 and 2016 in Freising, Germany (48°24′12.2″N, 11°43′22.3″E) with 419, 358, and 415 l m^−2^ rainfall from May to September (sowing to harvest), respectively. A drought stress experiment was carried out in 2016 in a rain-out shelter in Freising, Germany, (48°24′40.9″N, 11°43′22.4″E). Total irrigation from May to September 2016 in the rain-out shelter was 138 l m^−2^. The recurrent parent and the two NILs were part of larger trials, which were laid out as randomized complete block designs with two replications per entry in the field and three replications per entry in the rain-out shelter. The recurrent parent was included as triplicate entry in 2014 and duplicate entry in 2015 and 2016. NIL A was not included in the trial in 2015. NIL B was included as a duplicate entry in 2014. Each entry was planted in a single 1.2 m row with a 0.75 m distance between rows and intra-row spacing of 0.12 m, aiming at a plant density of 11 plants m^−2^. Application of herbicides and fertilizer followed good agricultural practice. All cobs per row were harvested manually and dried for 2 weeks at 30 °C before shelling. Grains were used for analysis of δ^13^C.

### Measurement of carbon isotope composition in grains

An analysis of carbon isotope composition in grains was performed by Isolab GmbH, Schweitenkirchen, Germany. Per sample, approximately 250 grains were first ground in a rotor beater mill (model SR300, Retsch, Haan, Germany) to about 0.5 mm particle size and then about 1 g was transferred to a 2 ml reaction tube, dried over night at 60 °C and ground to fine powder in a mixer mill (Mixer Mill MM 400, Retsch, Haan, Germany). Three milligrams ground grain material was used per sample and four technical replications were used for each biological replicate. For a more detailed description of the procedure, see Werner and Roßmann (2015).

### WUE_plant_ experiment in the greenhouse

Whole-plant water use efficiency (WUE_plant_) was evaluated in an experiment adapted from Yang et al. (2016). Maize plants were subjected to progressive drought stress by withholding water for 5 weeks. Single seedlings were grown in small pots for 2 weeks after germination (up to developmental stage V3–V4) in a growth chamber (16/8 h day night [d/n], 25/20 °C d/n, 650 μmol m^−2^ s^−1^ photosynthetically active radiation [PAR], 75% relative humidity [RH]) and then transferred to 10 l pots, containing the same amount of sieved homogeneous soil (CL ED73 from Einheitserdewerke Patzer, Germany; particle diameter less than 15 mm) and soil water content (SWC, vol/vol) ~ 85%. The two NILs and the recurrent parent were part of a larger experiment, containing a total of 23 maize genotypes, each represented by five plants (recurrent parent included as duplicate entry), organized in a randomized complete block design. Plastic bags (PE flat bags 400 × 600 × 0.05 mm, Baumann Saatzuchtbedarf, Germany) were used to cover the surface of the pots to avoid soil water evaporation and no further watering was applied until the end of the experiment. Supplemental light was used during the experiment. Climate conditions were monitored (25–33 °C/19–20 °C d/n, 400 μmol m^−2^ s^−1^ PAR, 40% RH). SWC was determined gravimetrically, by weighing the pots every 3–4 days, and the amount of water consumed by each plant was calculated as the difference from the initial pot weight at the beginning of the experiment. The experiment was ended when all plants stopped growing (developmental stage V9–V10), started senescing, and had consumed all of the available water. At the end of the experiment, above-ground material was harvested for biomass determination after drying the material for 1 week at 60 °C to achieve constant weight. WUE_plant_ was calculated as the ratio dry biomass/consumed water at the end of the experiment. As evaluation of WUE_plant_ is destructive, we assessed in a pre-test whether dry biomass differed between lines in 2-week-old plants, grown under the same conditions in the growth chamber as the plants included in the trial. Mean dry biomass was approximately 0.6 g with no significant differences between genotypes.

### High-throughput phenotyping of leaf elongation rate

A greenhouse experiment (March–April 2014) was carried out in the platform *Phenodyn* in Montpellier, France (Sadok et al. 2007; https://www6.montpellier.inra.fr/lepse/M3P), that measures leaf elongation rate (LER) with a 15 min time definition to test the growth sensitivity of the genotypes to evaporative demand and soil water deficit. The recurrent parent and the two NILs were part of a larger experiment containing 22 maize genotypes, organized in a randomized complete block design. Two treatments were applied: well-watered conditions (soil water potential, Ψ, maintained between − 0.14 and − 0.18 MPa) and progressive water deficit (Ψ dropping to − 0.7 MPa). Each genotype was represented by two pots (each containing 3 individual plants) per treatment. The recurrent parent was included as triplicate entry. Plants were grown in polyvinyl chloride columns (0.23 m diameter and 0.4 m height) containing a 40:60 (vol/vol) mixture of filtered loamy soil (particle diameter ranging from 0.1 to 4 mm) and organic compost. Daily watering with a modified one-tenth-strength Hoagland solution was applied to plants in both treatments to maintain the same soil water potential. Supplemental light was used to keep the photoperiod at 12 h of light and 12 h of dark and photosynthetic photon flux density (PPFD) at more than 400 µmol m^−2^ s^−1^. The elongation rate of the sixth leaf was measured with rotational displacement transducers (601–1045 Full 360° Smart Position Sensor; Spectrol Electronics) when the tip of the sixth leaf appeared above the whorl until leaf 8 appeared. LER values were expressed in millimeter per unit thermal time, calculated in equivalent hours at 20 °C as in Parent et al. (2010). Soil water content was measured by weighing the columns automatically every 15 min and a water release curve relating soil water content to predawn leaf water potential was used to estimate soil water potential over successive nights in the different conditions and individual pots. For the response to soil water status, values of LER for periods of 4 h at the end of the night were averaged and plotted against soil water potential. Air temperature and relative humidity were measured every minute at plant level (HMP35A, Vaisala Oy, Helsinki). The temperature of the meristematic zone of individual plants was measured with a fine copper-constantan thermocouple (0.2 mm diameter), located between the sheaths of leaves 1 and 2 at meristem height, to monitor deviations from the air temperature. Leaf-to-air vapour pressure was estimated at each time step as the difference in water vapour pressure between saturation at meristem temperature and the current vapour pressure in the air. Leaf-to-air vapour pressure did not differ between genotypes in well-watered conditions. Data analysis was performed using appropriate R scripts (R Core Team 2011; https://www.R-project.org). For a more detailed protocol, see Caldeira et al. (2014).

### Gas exchange measurements

Photosynthetic parameters, such as stomatal conductance (*g*_*s*_), net CO_2_ assimilation (*A*), evaporation (*E*), intracellular (*C*_*i*_), and atmospheric (*C*_a_) CO_2_ concentrations, were assessed in NIL B and the recurrent parent. Gas exchange measurements were performed on the last fully extended leaf on plants at V4–V5 developmental stage grown in a growth chamber (16/8 h d/n, 25/20 °C d/n, 75/70% RH d/n, max. 650 µmol m^−2^ s^−1^ PAR), using the GFS-3000 gas exchange system and its provided software (Heinz, Walz GmbH, Effeltrich, Germany) and on plants at V7–V8 developmental stage (10 biological replicates per genotype) grown in a greenhouse (25–33/19–20 °C d/n, 400 μmol m^−2^ s^−1^ PAR provided by supplemental light, 40% RH), using the portable photosynthesis system LI-6800 (LI-COR Inc., Lincoln, NE, USA). At both developmental stages, measurements were performed with CO_2_ concentration and temperature in leaf chamber kept at 400 μmol mol^−1^ and 25 ± 1 °C, respectively. Photon flux density was maintained at 1500 μmol m^−2^ s^−1^ by a red-blue light-emitting diode (LED) light source and at ambient RH. Intrinsic *i*WUE was calculated as the ratio between net CO_2_ assimilation and stomatal conductance (*A*/*g*_*s*_).

### Stomatal density

Stomatal density was evaluated in NIL B and the recurrent parent on plants grown in the same conditions in the growth chamber as those used for the gas exchange measurements. Nail varnish imprints were taken at three different places from both sides of the middle vein at the abaxial side in the middle of the fully expanded leaf 4 from maize plants at developmental stage V4–V5 (at least 8 biological replicates per genotype) and were immobilized on the surface of a microscopic slide with a cellophane transparent tape. No cover glass was used. Pictures were taken under a microscope (AxioPhot, Zeiss, Germany) with 10 × magnification at three different places of each imprint. Stomata were counted using ImageJ (bundled with Java 1.8.0_112, National Institutes of Health, USA) and their number per leaf area was calculated, using a microscopic scale under the same magnification.

### Leaf ABA quantification

Differences in abscisic acid (ABA) levels between NIL B and the recurrent parent were assessed in fully expanded maize leaves of plants at developmental stage V7–V8. ABA was quantified by using gas chromatography–mass spectrometry. The detailed protocol is provided in Online Resource 1.

### Statistical analyses

Analyses of variance (ANOVA) were performed for δ^13^C, measured in the field trials for each year and each treatment separately, as well as for the traits WUE_plant_, final dry biomass, SWC, and consumed water, measured in the WUE_plant_ trial, using the software PLABSTAT (Utz 2011; version 3A). For the comparisons between the recurrent parent and each of the two NILs, appropriate Student’s *t*-tests, accounting for multiple entries of recurrent parent and NILs in the field and greenhouse trials were conducted. Differences between the regression slopes of the three genotypes with respect to nighttime response of leaf elongation rate to decreasing soil water potential were tested with an ANOVA fitting the full model with different intercepts and slopes for each genotype and a reduced model with a common intercept and slope. Data analysis was performed using R (R Core Team 2011; https://www.R-project.org).

## Results

### Characterization of near-isogenic lines

**Fig. 1:**
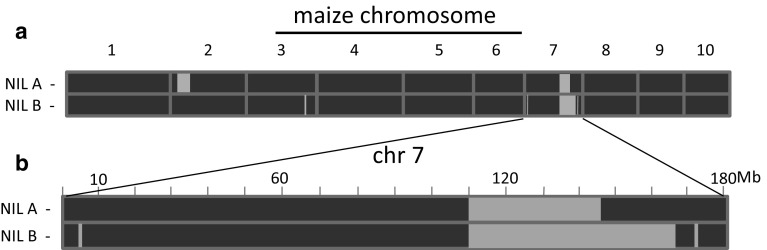
**a** Genomic composition of two near-isogenic lines, NIL A and NIL B, based on 616,201 SNP markers and **b** overlapping introgression segments on maize chromosome 7 between the two lines. Homozygous alleles of the donor parent (DP) are presented in green, homozygous alleles of the recurrent parent (RP) in blue (colour figure online)

The material under study originates from an introgression library described by Gresset et al. (2014). A detailed genotypic analysis with the 600 k Axiom™ Maize Genotyping Array (Unterseer et al. 2014) showed that NIL A and NIL B carry a genomic segment derived from the flint donor line on chr 7, which was shown to significantly increase grain δ^13^C compared to the recurrent parent. The length of the introgressed chr 7 segments in the two NILs is smaller compared to those of the lines from the original introgression library and the number and size of additional introgressions on other chromosomes is reduced (Fig. 1a).

From the data of Gresset et al. (2014) the chr 7 target region carrying a quantitative trait locus (QTL) on grain δ^13^C can be estimated to be 21.62 Mb long (located between 122.90 and 144.52 Mb; B73 v4 coordinates, available at www.maizegdb.org). NIL A and NIL B carry introgressions located between 110.76–146.67 and 110.76–166.10 Mb, respectively (Fig. 1b), including the target region, and only a few non-overlapping introgressions on chr 2 (NIL A), chr 3 (NIL B) and chr 7 (NIL B; Fig. 1a; Table S1, Online Resource 1).

### Introgression on chromosome 7 affects δ^13^C in maize under field conditions

Results from the field trials showed a significant increase in grain δ^13^C in control conditions for the two NILs compared to the recurrent parent (for NIL A only in 2014; Table 1). The difference between NIL B and the recurrent parent was larger than between NIL A and the recurrent parent. Hence, the increase in δ^13^C associated with introgressions on chr 7 under well-watered conditions (Gresset et al. 2014) can be considered as a reproducible phenotype. In 2015 and 2016, the rain-fed field had less precipitation around flowering time than in 2014 (Fig. S1). This is mirrored in the lower values for δ^13^C for all the lines in 2015 and 2016 compared to 2014. In water-deficit conditions, an overall decrease in δ^13^C was observed compared to control conditions with no significant differences between the NILs and the recurrent parent. ANOVA results are presented in Table S2 (Online Resource 1).

**Table 1:** Comparison of two near-isogenic lines (NIL A and NIL B) to their recurrent parent (RP) for carbon isotope composition (δ^13^C)

Years	Condition	δ^13^C[‰] per genotype		
		RP	NIL A	NIL B
2014	Control	− 12.34 ± 0.06	− 12.05 ± 0.11*	− 12.07 ± 0.08**
2015	Control	− 13.25 ± 0.06	NA	− 12.95 ± 0.08**
2016	Control	− 12.92 ± 0.05	− 12.83 ± 0.08	− 12.66 ± 0.08**
2016	Drought	− 13.43 ± 0.07	− 13.40 ± 0.10	− 13.25 ± 0.01

*NA* Not assessed

The trait was measured in field trials for three sequential years (2014, 2015, 2016) in well-watered (control) conditions and one year (2016) in water-limited conditions. Data are means ± SE (see “Materials and methods”). Significant differences between NIL A and NIL B with the recurrent parent are indicated with * (*P *< 0.05) and ** (*P *< 0.01), respectively

### Introgression on chromosome 7 causes higher sensitivity of growth to evaporative demand and soil water status

**Fig. 2:**
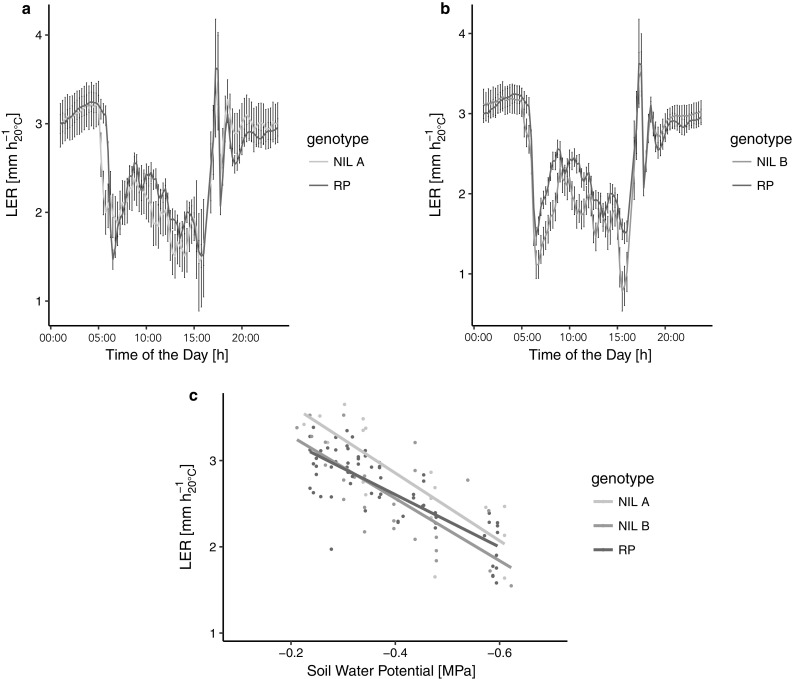
Leaf elongation rate (LER) of two near-isogenic lines, NIL A and NIL B, and their recurrent parent (RP). **a**, **b** LER response to change in evaporative demand during the day. Data are means ± confidence intervals at *P *< 0.05 (*n* = 6–56), **c** nighttime LER response to decreasing soil water potential. Data are means

We tested the growth sensitivity to evaporative demand and water deficit of the NILs and their recurrent parent in the *Phenodyn* platform. During the night, the values of evaporative demand were considered close to zero because of stomatal closure. During the day, they were inferred from the leaf-to-air vapour pressure difference that changed with time of day and was maximum around noon. LER was similar during the night in the three lines (Fig. 2a, b). It decreased rapidly at dawn, as previously observed in other lines (Caldeira et al. 2014), with a larger decrease in NIL B compared to the recurrent parent (Fig. 2b). The same was observed, to a lesser extent, in NIL A (Fig. 2a). Hence, leaf elongation rate was more sensitive to increase in evaporative demand in the NILs than in the recurrent parent.

During progressive soil drying, the slopes of the response of nighttime LER to soil water potential were significantly steeper (*P *< 0.01) in the NILs than in the recurrent parent (Fig. 2c), indicating a higher sensitivity of leaf elongation rate to soil water potential. Hence, leaf expansion was more sensitive to both evaporative demand and soil water potential in the NILs compared to the recurrent parent.

### Introgression on chromosome 7 decreases whole-plant water use efficiency

**Fig. 3:**
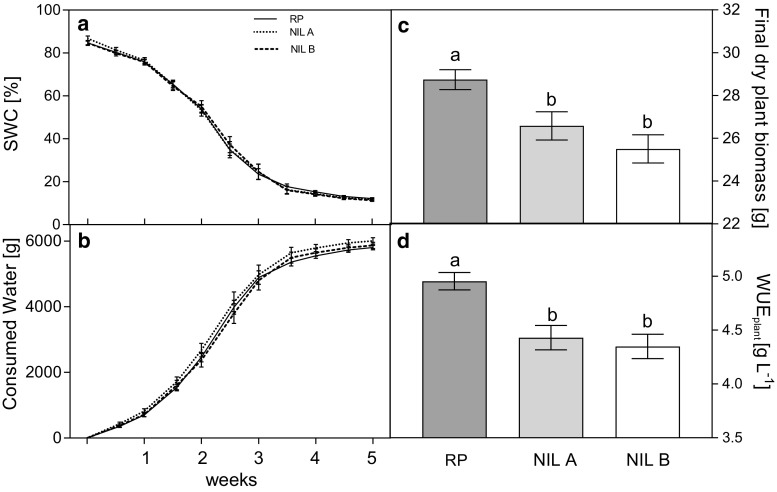
Comparison of two near-isogenic lines, NIL A and NIL B, and their recurrent parent (RP) under progressive soil drying conditions. Soil water content (SWC **a**) and consumed water (**b**) were monitored every 3–4 days. Final dry biomass **(c)** was measured at the end of the experiment and whole plant water use efficiency (WUE_plant_ **d**) was calculated as the ratio between final dry biomass and consumed water. No significant differences were measured between genotypes in initial mean dry biomass and it was therefore not included in the final WUE_plant_ calculation. Data are means ± standard error (*n* = 5). Significant differences between the lines based on Student’s *t*-test (*P *< 0.05) are indicated with different letters. RP values were already published (Blankenagel et al. 2018); however, they are included as a reference in the current study

To examine the association between δ^13^C and WUE, the two NILs and the recurrent parent were tested for their efficiency in accumulating biomass as a function of transpiration. We subjected the lines to progressive soil dehydration due to irrigation arrest with minimized water evaporation from the soil surface. The overlapping curves of progressive SWC decrease during the experiment indicated that the three genotypes experienced the soil drying at the same time (Fig. 3a) and consumed all the available water in the pots by the end of the experiment (Fig. 3b).

Final above-ground biomass was assessed at the end of the experiment (Fig. 3c). The dry weights of both NILs differed significantly from that of the recurrent parent, but not from each other. As the same amount of water was available to every plant and transpiration was similar in the three lines (Fig. 3b), the lower biomass of the NILs compared to the recurrent parent reflected significantly lower WUE_plant_ (Fig. 3d).

### Introgression on chromosome 7 increases stomatal conductance

**Fig. 4:**
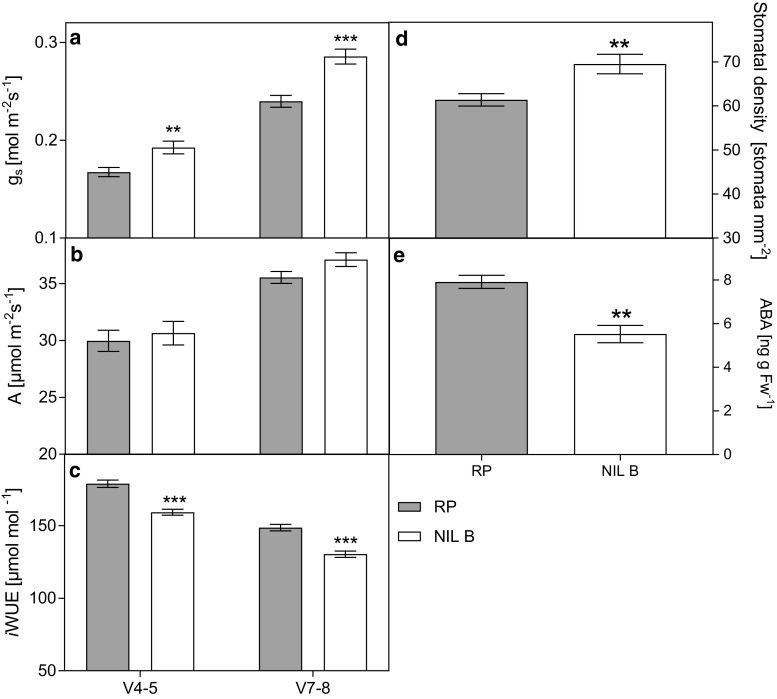
Comparison of the near-isogenic line NIL B and its recurrent parent (RP) for stomatal conductance (g_s_; **a**), net CO_2_ assimilation (**b**), intrinsic water use efficiency (*i*WUE; **c**), stomatal density (**d**) and leaf abscisic acid content (ABA; **e**). Measurements in **a**–**c** were done using an InfraRed Gas Analyzer (IRGA) at developmental stages V4–V5 in a growth chamber and V7–V8 in a greenhouse. Data are means ± standard error (*n* = 8–10). Significant differences, based on Student’s *t*-test, with *P *< 0.01, and *P *< 0.001 are marked with ** and ***, respectively

The whole-plant analysis reveals a similar transpiration in the NILs and in the recurrent parent, although leaf area is lower in the NILs due to lower leaf elongation rate. This suggests an increased transpiration per unit leaf area, which would also account for the reduced water use efficiency. A possible reason for higher transpiration per leaf area could be higher stomatal conductance. We have tested this hypothesis by measuring stomatal conductance and other photosynthetic parameters by gas exchange (Fig. 4 and Fig. S2, Online Resource 1). We chose NIL B over NIL A, because it has a lower percentage of donor parent genome (Table S1, Online Resource 1), no large segments on other chromosomes (Fig. 1), and differed from the recurrent parent to a greater extent with respect to most measured traits compared to NIL A. At two different developmental stages and in two different environments (V4–V5 in the growth chamber and V7–V8 in the greenhouse), NIL B showed significantly higher stomatal conductance (Fig. 4a) and consequently higher *E* (Fig. S2a), *C*_*i*_ (Fig. S2b), and *C*_*i*_ *C*_*a*_^−1^ (Fig. S2c) than the recurrent parent. No significant difference in CO_2_ assimilation (*A*) was detected in either of the two growth stages (Fig. 4b). Thus, intrinsic water use efficiency (*i*WUE), calculated as *A*/*g*_*s*_, was significantly decreased in NIL B compared to the recurrent parent (Fig. 4c). This was at least in part due to a higher stomatal density in NIL B compared to the recurrent parent (Fig. 4d). Higher stomatal conductance is a frequent cause of a lower WUE, due to the nonlinear relationship between photosynthesis and stomatal conductance (Farquhar et al. 1982b). As the phytohormone ABA is one of the main regulators of stomatal aperture and development in plant leaves (Chater et al. 2014), ABA content was measured in the fully developed leaf 7 (developmental stage V7–V8; Fig. 4e). Consistent with higher stomatal conductance and density in NIL B, ABA levels were significantly lower in this NIL than in the recurrent parent.

## Discussion

### A genomic segment on chromosome 7 affects several drought-related traits

Several physiological and genome based studies have provided valuable insights on the interplay and trade-off between drought-associated traits in maize (Welcker et al. 2011; Harrison et al. 2014; Yan et al. 2016), but even with sophisticated statistical methods at hand (e.g. Töpner et al. 2017), inferring the relation between traits behind genetic correlations is a non-trivial task. Here, we used near-isogenic lines to analyse the effect of a specific genomic segment, which affects carbon isotope discrimination and other drought-associated traits, in an otherwise homogeneous genetic background. Our two NILs contain less than 4% donor parent genome (Table S1, Online Resource 1) and are very similar to the recurrent parent with respect to important agronomic traits, such as flowering time, plant height, and thousand grain weight, under control and drought conditions in the field (data not shown). This is important, to exclude that the observed effects originate from morphological differences between the investigated lines.

The chr 7 segment affected δ^13^C, growth sensitivity to both evaporative demand and decrease in soil water content, as well as WUE_plant_, *i*WUE, *g*_*s*_, stomatal density, and leaf ABA content. Co-localized QTL for WUE and δ^13^C have previously been found in C_3_ species (Adiredjo et al. 2014; Easlon et al. 2014) and recently in the C_4_ species *Setaria* (Ellsworth et al. 2018). Welcker et al. (2011) showed for maize that responses to both evaporative demand and soil water deficit are affected to a large extent by the same genomic regions. Genetic co-localization of multiple traits may be due to pleiotropy or tight linkage, resulting from evolution or breeding programs selecting the traits simultaneously during plant improvement (Welcker et al. 2011). However, as pointed out by Gianola et al. (2015), making inferences on causes of correlations from genomic studies can only be conjectural. Based on our results with near-isogenic lines, we hypothesize that the introgression segment on chr 7 (110.76–166.10 Mb) carried by NIL B harbours several QTL that affect different traits and have a cumulative effect on individual traits. The latter can be inferred from NIL A with a smaller segment on chr 7 than NIL B and a less pronounced effect on the measured parameters. Additional evidence comes from a study of Alvarez Prado et al. (2018), who identified three neighbouring QTL affecting whole-plant stomatal conductance (two with positive and one with negative effect) in the same genomic region (124.35–160.14 Mb) on chr 7 in a maize diversity panel. Only two of them (with opposite effects on g_s_) are included in the smaller (110.76–146.67 Mb) introgression segment of NIL A. This might be one explanation for the weaker differences of NIL A than of NIL B compared with the recurrent parent.

On the other hand, NIL A carries a second large segment on chr 2 (Table S1, Online Resource 1), where a previously identified QTL for δ^13^C is located (Gresset et al. 2014), which might alter the effect of the introgression on chr 7. As this needs to be confirmed in further genetic studies, we decided to focus our analyses on NIL B, as it will allow the dissection of the entire chr 7 region, including all three QTL for g_s_ described by Alvarez Prado et al. (2018). Through genetic fine-mapping, it should be feasible to explore whether the genetic basis of the investigated traits is qualitative or quantitative. It should also be possible to elucidate whether the regulation of all the measured traits is due to the same physiological factor, such as hydraulic changes related to stomatal behaviour or due to different mechanisms, controlled by closely linked causal genes in the genomic region.

### Stomatal properties regulated by ABA most likely establish the connection between δ^13^C, WUE, and growth sensitivity to evaporative demand and soil water status

Our results suggest that, for the genetic material under study, stomatal properties may establish the connection between all measured traits. We show a positive association between δ^13^C and g_s_ in maize (Table 1 and Fig. 4a), observed also in other C_4_ species (Henderson et al. 1998; Dercon et al. 2006; Sharwood et al. 2014). A significant difference between NIL B and the recurrent parent in grain δ^13^C was observed only in well-watered conditions, in agreement with findings in the C_4_ species *Setaria* (Ellsworth et al. 2018), where differences in δ^13^C were small under water-limited conditions. The authors explain this with lower stomatal conductance leveling differences in transpiration efficiency and hence differences in δ^13^C between genotypes, like it was reported in C_3_ species (Adiredjo et al. 2014) and maize (Alvarez Prado et al. 2018). Differences in both δ^13^C and *g*_*s*_ between NIL B and the recurrent parent were stable over several field seasons for δ^13^C and over different developmental stages and environments for g_s_. However, it is important to point out that, even though we found an association between *C*_*i*_ *C*_*a*_^−1^ and δ^13^C, this does not mean that *g*_*s*_ is the main regulator of δ^13^C. In C_4_ plants, additional factors, such as activities of CO_2_-fixing enzymes (Rubisco, PEPC, carbonic anhydrase), leakiness, and mesophyll conductance (*g*_*m*_), could also be genotype-dependent and contribute to the observed variation in δ^13^C (Farquhar et al. 1982a; Saliendra et al. 1996, Barbour et al. 2010; von Caemmerer et al. 2014). Since grain δ^13^C is an integrated value, post-photosynthetic factors, such as respiration and photorespiration, could also play a role (Ubierna et al. 2013, von Caemmerer et al. 2014).

Stomatal conductance determines *i*WUE. However, due to diurnal changes in g_s_ and environmental conditions, *i*WUE can only be loosely related to WUE_plant_ which is measured over a long time period (Jones 2004). Hence, studying the relationship between *i*WUE and WUE_plant_, is important for a given species and growth condition in order to deduce to what extent stomatal conductance relates to plant productivity. The observed difference in g_s_ between NIL B and the recurrent parent is also associated with a difference in the amount of biomass built for a given volume of water transpired between the lines, i.e. WUE_plant_ (Figs. 3d and 4a). Similar observations have been made in *Setaria*, where correlations of fresh biomass, transpiration, and WUE have been established with leaf δ^13^C in a recombinant inbred line (RIL) population and co-localizing QTL were found for these traits in well-watered conditions (Ellsworth et al. 2018). Taken together, the findings in *Setaria* and our results in maize demonstrate a genetic and physiological association between δ^13^C and WUE in C_4_ species, which most probably involves regulation through stomatal conductance. We showed that, in maize, grain δ^13^C is both genetically determined and responsive to drought as it has recently been shown for leaf δ^13^C in *Setaria* (Ellsworth et al. 2018). Hence, our results suggest that, in spite of the fact that the underlying mechanisms are still not clear, δ^13^C can be used in maize as a proxy of other variables involved in drought tolerance. It remains to be shown whether these trait associations are exhibited also by other chromosomal regions and persist in diverse genetic material.

Although it is generally accepted that the sensitivity of leaf elongation rate to the two components of drought stress, evaporative demand and soil water deficit, depends on different physiological control mechanisms, g_s_ and hydraulic conductance are suggested to play a role in both responses (Welcker et al. 2011). Our results are in accordance with these findings, as NIL B and the recurrent parent differed significantly in their stomatal properties (Fig. 4a, d) and also in their growth sensitivity to both evaporative demand and soil water deficit (Fig. 2).

Stomatal behaviour is controlled by ABA by regulation of ion channels in guard cells and by indirect hydraulic effects (Pantin et al. 2013). It has also been shown that ABA biosynthesis mutants exhibiting reduced ABA levels have higher stomatal density (Tanaka et al. 2013). For NIL B, lower leaf ABA concentration and higher stomatal density at early developmental stages (V4–V8) compared to the recurrent parent could be shown (Fig. 4d, e). Therefore, our findings are in agreement with the hypothesis about the dual effect of ABA on stomata, by regulating not only their aperture, but also their development (Chater et al. 2014) and suggest that the observed differences in g_s_ and consequently in WUE and drought sensitivity could at least partially result from leaf anatomy. Similar results were observed in rice and wheat (Li et al. 2017; Ouyang et al. 2017).

In summary, our results show that a single genomic segment influences δ^13^C, leaf growth sensitivity to increase in evaporative demand and decrease in soil water potential, *i*WUE, and WUE_plant_. A reduction of leaf ABA content in the NIL compared to the recurrent parent suggests at least partial involvement of altered stomatal properties in the effect harboured in the chr 7 segment. The genetic material at hand provides a unique opportunity to disentangle the associations between carbon isotope composition, WUE, and stomatal properties at the genetic and physiological level.

## Electronic supplementary material

Below is the link to the electronic supplementary material.

Supplementary material 1 (PDF 277 kb)
