# Supplementary material for: Carbon isotope composition, water use efficiency, and drought sensitivity are controlled by a common genomic segment in maize
Source: Theor Appl Genet. 2018 Sep 22;132(1):53–63. doi: 10.1007/s00122-018-3193-4 (PMC6320357; doi:10.1007/s00122-018-3193-4)
Supplement: Supplementary file 1 — Supplementary material 1 (PDF 277 kb) [file 122_2018_3193_MOESM1_ESM.pdf]

Supplementary material

Theoretical and Applied Genetics

Online Resource 1

Title: Carbon isotope composition, water use efficiency, and drought sensitivity are controlled by a common genomic segment in maize

Authors: Viktoriya Avramova[1], Adel Meziane[2], Eva Bauer[1], Sonja Blankenagel[1], Stella Eggels[1], Sebastian Gresset[1], Erwin Grill[3], Claudiu Niculaes[1], Milena Ouzunova[4], Brigitte Poppenberger[5], Thomas Presterl[4], Wilfried Rozhon[5], Claude Welcker[2], Zhenyu Yang[3], François Tardieu[2], and Chris-Carolin Schön[1,*]

Author affiliations:

[1]Plant Breeding, TUM School of Life Sciences Weihenstephan, Technical University of Munich, Liesel-Beckmann-Straße 2, 85354 Freising, Germany

[2]INRA, UMR759 Laboratoire d'Ecophysiologie des Plantes sous Stress Environnementaux, Place Viala, F-34060, Montpellier, France

[3]Botany, TUM School of Life Sciences Weihenstephan, Technical University of Munich, Emil-Ramann-Straße 4, 85354 Freising, Germany

[4]KWS SAAT SE, Grimsehlstraße 31, 37555 Einbeck, Germany

[5]Biotechnology of Horticultural Crops, TUM School of Life Sciences Weihenstephan, Technical University Munich, Liesel-Beckmann-Straße 1, 85354 Freising, Germany

[*]Corresponding author

Contact information of the corresponding author:

Phone: +49 816171 3422

E-mail address: chris.schoen@tum.de

Table S1 Pedigree of two nearly isogenic lines (NILs) and regions with genomic introgressions from a donor parent (DP, an elite flint line) in the background of a recurrent dent parent (RP, an elite dent line).

NIL	Pedigree	Genomic flint introgressions	Total DP genome (%)
NIL A<br>(original name: subIL_10687_2)	IL_015 x IL_59 (Gresset et. al, 2014)	Chr 2: 20.39-66.18 Mb<br>Chr 7: 110.76-146.67 Mb	3.55
NIL B<br>(original name: subIL_10860_2)	IL_027 x IL_59 (Gresset et. al, 2014)	Chr 3: 212.55-218.25 Mb<br>Chr 7: 4.08-5.30 Mb; 110.76-166.10 Mb; 172.48-173.53 Mb	2.75

Table S2 Analysis of variance for the trait carbon isotope composition ($\delta^{13}C$), measured in field trials in the years 2014, 2015 and 2016 and for two treatments (control, **a**, **b**, and **c**, and drought treatment, **d**).

a) 2014 control treatment

**Source of variance**	**DF**	**Mean Square**	**Variance components**	**F-value**	**Significance**
Replications	1	0.0287	0.0001	1.20	
Treatment	56	0.4419	0.2090	18.42	**
Intra-block error	52	0.0240	0.0240		

Significance codes: 0.001 "***"; 0.01 "**"; 0.05 "*"

b) 2015 control treatment

**Source of variance**	**DF**	**Mean Square**	**Variance components**	**F-value**	**Significance**
Replications	1	0.0447	0.0008	3.19	
Treatment	34	0.1497	0.0679	10.69	**
Intra-block error	33	0.0140	0.0140		

Significance codes: 0.001 "***"; 0.01 "**"; 0.05 "*"

c) 2016 control treatment

**Source of variance**	**DF**	**Mean Square**	**Variance components**	**F-value**	**Significance**
Replications	1	0.0008	-0.0002	0.07	
Treatment	53	0.1918	0.0900	16.26	**
Intra-block error	43	0.0118	0.0118		

Significance codes: 0.001 "***"; 0.01 "**"; 0.05 "*"

d) 2016 drought treatment

**Source of variance**	**DF**	**Mean Square**	**Variance components**	**F-value**	**Significance**
Replications	2	0.1390	0.0019	4.36	*
Treatment	55	0.2312	0.0664	7.25	**
Intra-block error	105	0.0319	0.0319		

Significance codes: 0.001 "***"; 0.01 "**"; 0.05 "*"

Fig. S1 Daily precipitation (bars, [l/m^2]) and average temperature (line, [°C]) in **a**) Roggenstein 2014 (control), **b**) Freising 2015 (control), **c**) Freising 2016 (control), **d**) Freising 2016 (rain-out shelter).

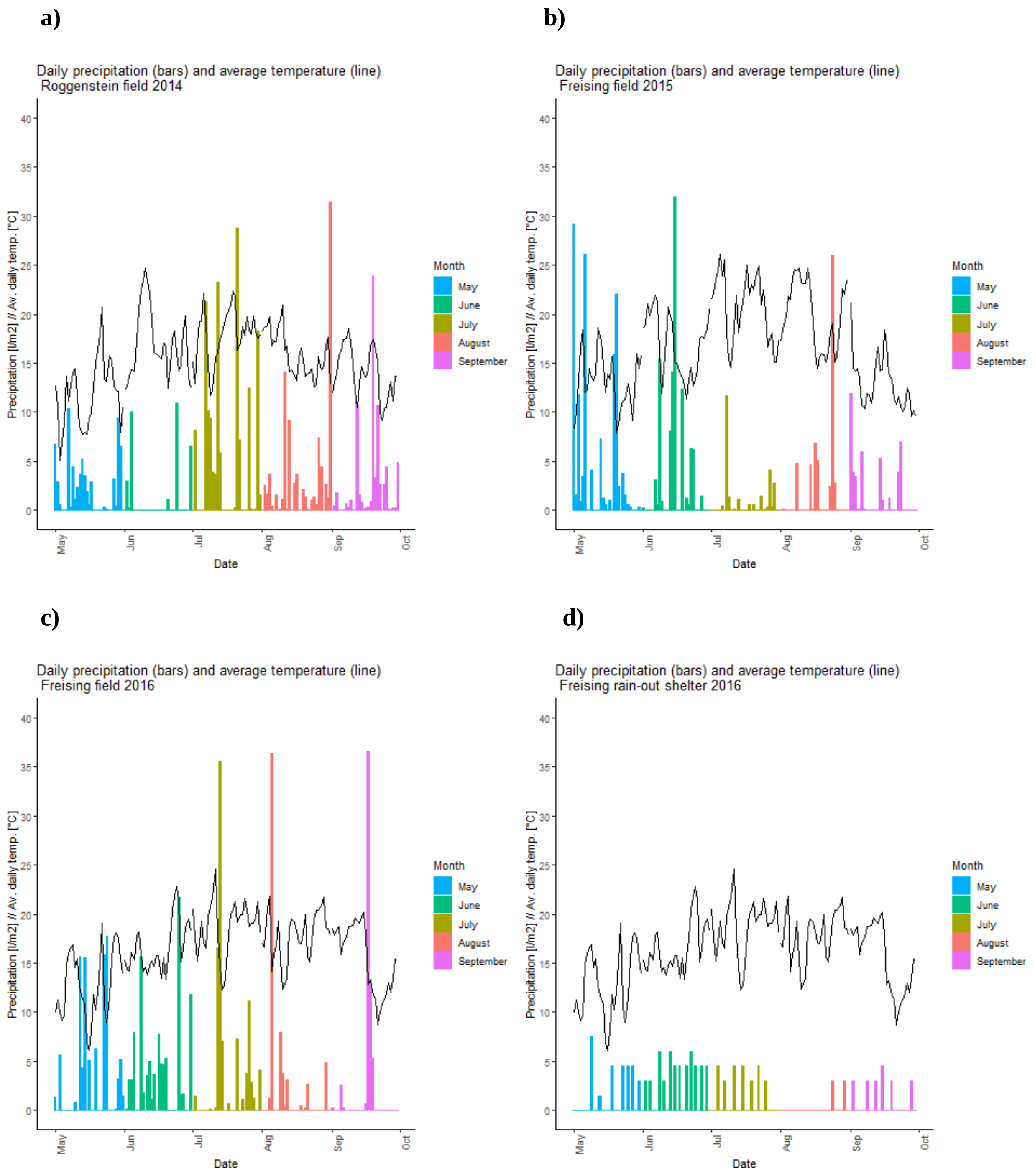

Fig. S2 Comparison of the near isogenic line NIL B and its recurrent parent (RP) for leaf evaporation (E; **a**), intracellular CO_2 concentration (C_i; **b**), and the ratio of intracellular and atmospheric CO_2 concentration ($C_i\ C_a^{-1}$; **c**). Data were measured by an InfraRed Gas Analyzer (IRGA) at developmental stages V4-V5 in a growth chamber and V7-V8 in a greenhouse. Data are means ± standard error (n=8-10). Significant differences, based on Student's t-test, with P<0.05, and P<0.001 are marked with * and ***, respectively.

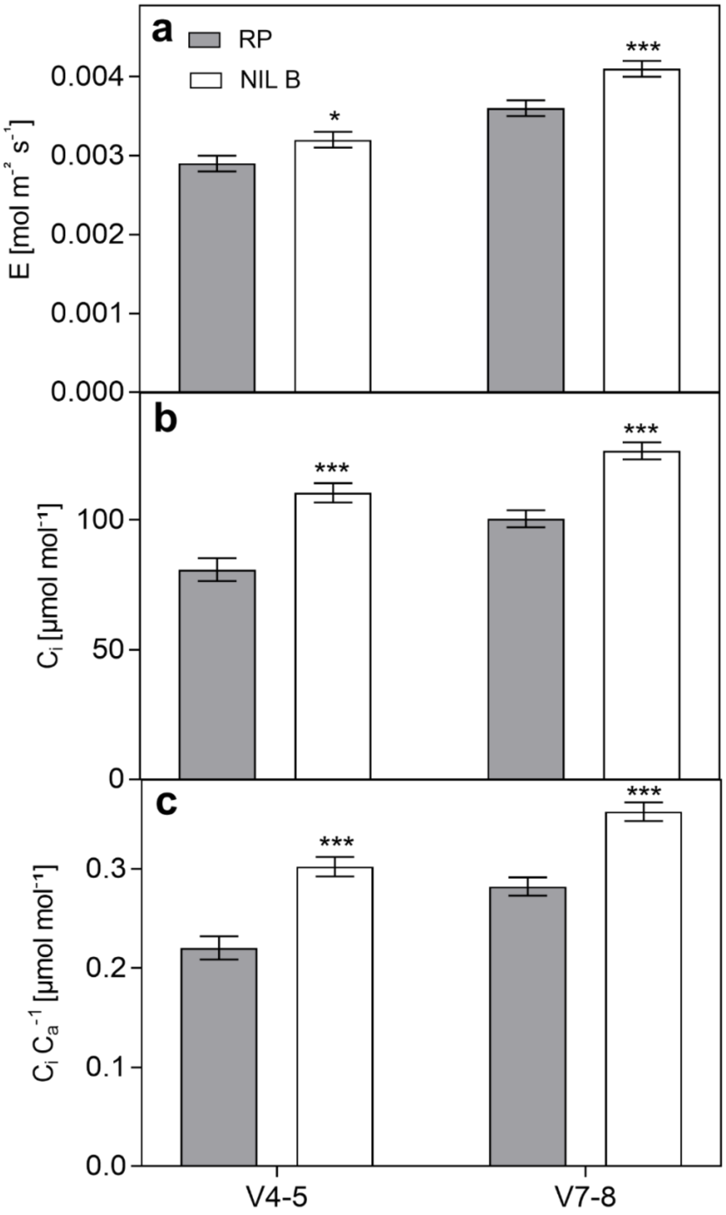

Quantification of ABA

The frozen, finely powdered plant material (1.5 to 2 g) was transferred into a 50 ml screw cap tube and 100 µl internal standard (ABA-d_6, 15 ng/100 µl) was added. 15 ml 40% acetonitrile (ACN) and 500 µl sodium phosphate buffer (0.5 M, pH 7.0) were added to the plant material and the mixture incubated on an orbital shaker at 350 rpm for 60 min. 500 µl 4 M phosphoric acid and 10 ml tert-butylmethylether were added and the mixture was shaken on an orbital at 350 rpm for 30 min. Phases were separated by centrifugation at 4000×g for 5 min and the upper phase was transferred to a new tube and mixed with 5 ml 100% ACN. The lower, aqueous phase was again extracted with 10 ml tert-butylmethylether as described above and the upper phase pooled with the previous one. The combined organic phases were passed over a Chromabond NH2 500 mg solid phase extraction column (Macherey Nagel, Düren, Germany) that had been equilibrated with 3 ml 100% ACN. The column was washed with 3 ml 100% ACN prior elution with 3 ml 1 M phosphoric acid in 20% ACN. The eluate was diluted with water to a final volume of 14 ml and passed over a Chromabond C18ec 100 mg solid phase extraction column (Macherey Nagel) that had been equilibrated two times with 1 ml 100% ACN and once with 1 ml water. The column was washed with 1 ml 10 mM phosphoric acid prior elution with 1 ml 80% ACN. The eluate was evaporated to dryness in a vacuum concentrator. The residue was dissolved in 100 µl 100 mM phosphoric acid in 20% ACN. The solution was fractionated by RP-HPLC using a Nucleodur 100-5 C18ec 125 x 4.6 mm column and isocratic elution with 27% ACN containing 1 mM formic acid at a flow rate of 0.8 ml/min. The ABA-containing fraction with a retention time from 10 to 11.2 min was collected and evaporated in the vacuum to dryness. The residue was methylated by addition of 100 µl of a mixture consisting of (trimethylsilyl)diazomethane solution (2 M in diethyl ether; Sigma Aldrich, St. Louis, MO, USA) and methanol at a ratio of 1 to 19. The reaction was incubated with gentle agitation at 25°C for 10 min prior evaporation under vacuum. The residue was dissolved in 20 µl 100% ACN and analyzed by GC-MS using a 431-GC gas chromatograph equipped with a CP-8400 autosampler and connected to a 210-MS mass spectrometer (Agilent, St. Clara, CA, USA). The injection volume was 7 µl. A VF-5ms 30 m x 0.25 mm column with 25 µm film thickness (Agilent) was used for separation. The column oven temperature was initially kept at 120°C for 2 min and then increased at a rate of 10°C/min to 190°C. Next, the temperature was increased at a rate of 0.7°C/min to 199°C. Finally, the temperature was raised to 320°C with a rate of 40°C/min. The ion trap was operated at 180°C, the transfer line at 200°C and the manifold at 40°C. MS spectra were recorded from a retention time

of 18 to 21 min. The masses of 190 (ABA) and 194 (internal standard) were used for quantification. The masses of 134 and 162 for ABA and of 138 and 166 for the internal standard were used for confirmation of identity.
